# Multimodal Biometric Framework for Evaluating Emotional Impact of Chromatic Manipulation in Cinematic Content

**DOI:** 10.3390/s26113349

**Published:** 2026-05-25

**Authors:** Carolina Del-Valle-Soto, Juan Arturo Nolazco-Flores, Jesus GomezRomero-Borquez, Andres Gonzalez-Gomez, Martin Garcia-Torres, Violeta Corona, Juan-Carlos López-Pimentel, Paolo Visconti

**Affiliations:** 1 Facultad de Ingeniería, Universidad Panamericana, Álvaro del Portillo 49, Zapopan 45010, JA, Mexico; jagomez@up.edu.mx (J.G.-B.); 0255690@up.edu.mx (A.G.-G.); 0254900@up.edu.mx (M.G.-T.); clopezp@up.edu.mx (J.-C.L.-P.); 2 School of Engineering and Science, Tecnológico de Monterrey, Av. Eugenio Garza Sada 2501, Monterrey 64849, NL, Mexico; jnolazco@tec.mx; 3 Facultad de Ciencias Económicas y Empresariales, Universidad Panamericana, Álvaro del Portillo 49, Zapopan 45010, JA, Mexico; vcorona@up.edu.mx; 4 Department of Innovation Engineering, University of Salento, 73100 Lecce, Italy; paolo.visconti@unisalento.it

**Keywords:** affective computing, chromatic manipulation, facial expression analysis, AFFDEX, galvanic skin response, latin-square design, permutation test, cluster bootstrap, multimodal emotion recognition, sex differences

## Abstract

This study investigates how chromatic manipulation of cinematic content modulates emotional engagement, with specific attention to sex-differentiated responses. We used a mixed factorial design with chromatic condition as a within-subject factor and biological sex as a between-subject factor, counterbalanced across scenes through a 3 × 3 Latin square that renders scene identity orthogonal to chromatic condition by construction. Thirty adult viewers were recorded with synchronised facial-expression analysis (AFFDEX 5.1), blink detection, and galvanic skin response (Shimmer GSR). The primary inferential target was the Condition × Sex interaction on automated positive facial valence. This interaction was statistically reliable under three converging tests: a mixed-effects model ($\beta_{\mathrm{Mod}\times F}=-4.48$, SE=2.16, 95% CI [−8.81,−0.14], p=0.043), a participant-level cluster bootstrap (2000 resamples; 95% percentile CI [−9.78,−0.63]; $p_{\mathrm{boot}}=0.011$), and a label-permutation test. The effect was stable under leave-one-subject-out resampling (100% sign-stability) and persisted after introducing scene as a fixed factor. Blink rate and electrodermal activation showed directionally consistent but weaker interaction patterns. A multidimensional engagement framework that separates attentional-autonomic intensity from expressive valence supports interpretation of the finding as specific to expressive affective behavior rather than to overall activation. The results provide empirical evidence that chromatic manipulation in realistic cinematic stimuli modulates expressive affective responses in a sex-dependent manner, and they establish a reproducible multimodal biometric framework for chromatic impact assessment.

## 1. Introduction

Color in cinema is widely recognized as a powerful narrative tool that shapes emotional and perceptual experiences. While it can evoke mood, convey symbolism, and guide attention, its emotional potential is often underutilized, leading to narrative dissonance when chromatic choices lack psychological coherence [1]. This study examines how chromatic variation, across original, black-and-white, and color-manipulated versions of film scenes, affects emotional perception using multimodal biometric tools such as facial expression analysis (AFFDEX 5.1) and galvanic skin response sensors (Shimmer GSR), with attention to sex-based differences [2,3]. Grounded in color psychology and cinematic theory [4], the research frames color as a deliberate emotional modulator. It aligns with neurocinematics and affective computing, fields that explore how visual stimuli, including color, can trigger synchronized affective responses and be measured in real time [5,6]. Prior research in color psychology suggests systematic associations between chromatic attributes and affective dimensions such as arousal and valence. Controlled experimental studies have reported that warm hues (e.g., red, orange) are often associated with higher perceived arousal, whereas cool hues (e.g., blue, green) tend to be linked to lower arousal or calming effects; however, these associations are probabilistic rather than deterministic and are strongly moderated by contextual, cultural, and task-related factors [7,8]. Importantly, much of the existing literature relies on static or decontextualized stimuli, which may not generalize directly to dynamic cinematic environments. Accordingly, chromatic influences on emotional response should be interpreted as context-dependent modulations rather than universally replicable effects. Bridging artistic and scientific domains, the study proposes a rigorous, data-driven framework to inform emotionally aware design in film, interactive media, and virtual environments [9].

Advances in cognitive science and media psychology have demonstrated that color perception is closely linked to affective processing, influencing both subjective emotional appraisal and measurable physiological responses [10]. Experimental research in color psychology suggests that perceptual dimensions such as hue and saturation can influence affective appraisal along arousal and valence axes, although effect sizes are typically moderate and sensitive to stimulus configuration [7]. Importantly, cross-cultural investigations and environmental psychology studies demonstrate that color–emotion associations are neither universal nor fixed, but are moderated by contextual framing, semantic meaning, cultural background, and task demands [8,11]. Such variability underscores the importance of distinguishing between laboratory-based color perception studies and emotionally embedded audiovisual experiences. In narrative media contexts, these moderating factors may interact with genre conventions, character identification, and scene dynamics, underscoring the need for ecologically valid experimental paradigms that extend beyond static or decontextualized stimuli.

Importantly, affective responses to color in dynamic visual contexts may arise from multiple interacting mechanisms. In addition to higher-level narrative and semantic processing, color manipulations may also induce low-level perceptual effects related to chromatic salience, novelty, or deviation from expected color distributions. Experimental evidence suggests that human observers exhibit differential sensitivity to chromatic variation, with some studies reporting sex-related differences in color discrimination and perceptual processing efficiency [12,13]. Accordingly, physiological reactivity to chromatic manipulation may reflect not only narrative-emotional engagement but also perceptual responses to visual irregularity or “color oddity.” This distinction is particularly relevant when color transformations deviate from ecologically typical or cinematographically coherent palettes.

In parallel, the emergence of neurocinematics and affective computing has opened new avenues for the objective study of emotional engagement during audiovisual experiences. These fields investigate how dynamic visual stimuli can elicit synchronized emotional and physiological responses, measurable in real time using biometric and behavioral signals [14,15]. Within this framework, multiple physiological and behavioral indicators have been investigated as correlates of attentional allocation and affective processing. Electrodermal activity is widely regarded as a robust index of sympathetic arousal due to its direct linkage to eccrine sweat gland activity [16,17]. Eye-blink rate and suppression have been associated with fluctuations in attentional load and cognitive engagement, particularly in dynamic visual tasks [14,18]. Similarly, automated facial expression analysis systems have demonstrated sensitivity to affective valence and discrete emotion-related facial action units. However, their outputs should be interpreted as probabilistic estimates rather than direct measures of internal emotional states, particularly in naturalistic contexts. Empirical evidence indicates that the accuracy of such systems decreases when detecting spontaneous, low-intensity expressions compared with prototypical posed expressions, often resulting in a bias toward neutral classifications and an underestimation of subtle affective responses [19,20]. This limitation is especially relevant in passive viewing scenarios such as cinematic exposure, where emotional responses are typically expressed through micro-expressions and low-amplitude facial movements rather than overt configurations. Together, these measures provide complementary but non-redundant indicators of affective and attentional dynamics. Importantly, such measures allow for the continuous, unobtrusive quantification of emotional responses, preserving ecological validity while enabling rigorous analysis.

Engagement should not be reduced to a single affective dimension. Contemporary affective science frameworks conceptualize emotional experience along at least two partially independent dimensions: arousal (reflecting intensity of activation) and valence (reflecting the hedonic direction of the experience) [21,22].

Although prior work has explored the relationship between color and emotion, empirical studies that integrate chromatic manipulation with multimodal biometric sensing in cinematic contexts remain limited. A substantial portion of empirical research on color and emotion has relied on static, decontextualized stimuli (e.g., color patches or still images) and post-exposure self-report measures of affect. While such paradigms offer experimental control, they may limit ecological validity by failing to capture the temporal dynamics, narrative embedding, and multisensory integration inherent in cinematic experiences [7,23]. Moreover, exclusive reliance on subjective evaluations introduces potential biases related to introspective access, demand characteristics, and inter-individual differences in emotional reporting [24]. Importantly, accumulating evidence suggests that individual difference variables, including biological sex, can moderate both emotional expressivity and autonomic reactivity, indicating that affective responses to audiovisual stimuli are not uniformly distributed across populations [25,26]. Failure to account for such moderating factors may obscure interaction effects and reduce the explanatory power of experimental models. These methodological considerations underscore the need for multimodal, ecologically valid approaches capable of capturing both physiological and expressive components of affect within dynamic narrative contexts. Consequently, there is a methodological gap between theoretical accounts of color as an emotional modulator and reproducible frameworks capable of evaluating its impact under realistic viewing conditions.

To address this gap, the present study investigates how chromatic variation, specifically original color, black-and-white, and algorithmically manipulated color versions of cinematic scenes, affects emotional perception as measured through a multimodal biometric approach. Facial expression analysis using AFFDEX 5.1 and galvanic skin response sensing via Shimmer GSR are employed to capture complementary dimensions of affective engagement, including expressive behavior and autonomic activation. The inclusion of biological sex as a between-subjects factor is theoretically motivated by evidence from affective neuroscience and psychophysiology indicating sex-related differences in emotional processing, autonomic reactivity, and expressive behavior [25,26]. Prior research suggests that women, on average, exhibit greater emotional expressivity and heightened electrodermal responsiveness to affective stimuli, particularly in socially salient or relational contexts. From a theoretical perspective, if chromatic modulation alters affective salience or perceptual–emotional coupling, its impact may be moderated by sex-linked differences in sensory-affective integration and autonomic regulation. Therefore, sex is incorporated not as a descriptive demographic variable, but as a theoretically grounded moderator within the experimental design. This approach aligns with interactionist models of emotion, which posit that stimulus properties and observer characteristics jointly determine affective outcomes.

The primary contribution of this work lies in the integration and validation of a multimodal biometric evaluation framework for chromatic impact assessment, rather than in narrative interpretation itself. Although affective computing has developed robust techniques for emotion recognition across diverse domains, including human–computer interaction, virtual reality, and multimedia analysis [27,28], their application to cinematic and narrative media has often been either modality-specific or focused on global classification of emotional states rather than on experimentally controlled manipulation of audiovisual parameters.

In contrast, this work is designed to evaluate the causal impact of controlled chromatic manipulation within dynamic cinematic stimuli using an integrated, multimodal biometric approach. Rather than merely detecting emotion, the proposed methodology aims to model how specific visual design variables interact with physiological and expressive indicators under a factorial experimental structure. This distinction is critical because narrative media involve temporally evolving, contextually embedded stimuli that cannot be adequately characterized through static-image paradigms or single-signal inference models.

By bridging artistic practice with empirical measurement, this study aims to inform emotionally aware design strategies in film, interactive media, and virtual environments, contributing both methodological tools and experimental evidence to the study of affective visual communication [9].

While multimodal biometric approaches to emotion recognition are well established in affective computing and media research [27,29], relatively few studies have isolated a single cinematic design variable, such as chromatic composition, within a controlled factorial structure while preserving the temporal and narrative continuity of film stimuli.

### Motivation

This experimental research addresses a critical gap in both scholarly and practical domains concerning the emotional use of color in cinematographic production, especially in animation. Although color is widely recognized for its ability to evoke emotions and guide narrative tone, it is often applied in ways that prioritize aesthetics over emotional effectiveness, leading to a mismatch between the intended mood and the viewer’s actual emotional response. The study investigates whether modifications in saturation, temperature, and contrast within cinematic scenes influence emotional perception, an especially relevant question in animation, where every visual element is deliberately crafted. The central hypothesis of the study is that systematic alterations of chromatic parameters (hue, saturation, and temperature) that deviate from the original cinematographic grading may modulate the coherence between narrative valence and viewers’ physiological-expressive responses. Specifically, we test whether experimentally induced chromatic shifts produce measurable changes in electrodermal activation, blink dynamics, and facial expression patterns relative to the original color condition, despite identical narrative content. Under this formulation, “emotional alignment” is operationalized as the degree of correspondence between the intended affective tone of a scene and multimodal biometric indicators of arousal and valence.

Formally, the primary hypothesis tested in this study is:

**H1.**  *Under controlled cinematic exposure with scene identity orthogonalized to chromatic condition, the Condition × Sex interaction on automated positive facial valence is reliably different from zero*.

This formulation makes the inferential target explicit (an interaction coefficient), specifies the design-level control (Latin-square counterbalancing of scenes), and identifies the primary dependent variable (positive facial valence from AFFDEX 5.1). The two secondary dependent variables, blink rate and galvanic skin response peaks per minute, are analyzed under the same statistical framework but treated as complementary attentional/autonomic indicators rather than as primary tests of H1.

The remainder of this article is structured as follows. Section 2 reviews related work on color psychology, neurocinematics, and multimodal affective inference. Section 3 describes the experimental design, including the Latin-square counterbalancing and biometric instrumentation. Section 4 specifies the statistical modeling framework, including the non-parametric inference procedures. Section 5 reports the main results, Section 6 discusses their implications and limitations, and Section 7 concludes.

## 2. Related Work

Recent advances in affective computing and neurocinematics have shown that visual elements like color, luminance, and contrast significantly influence emotional engagement in film. Cross-cultural studies confirm that red is often linked to arousal and urgency, while blue is associated with calmness and introspection [30]. Chromatic features, such as saturation and temperature, also affect physiological responses, as measured by GSR, and visual attention patterns, though their emotional effects are context-dependent and shaped by genre and cultural background [31]. Notably, the expression of joy, particularly the AU12 (smile) action unit in FACS, occurs more often in color-rich scenes, especially those using warm hues like yellow, which intensify emotional resonance [8]. As Jonauskaite highlights, color modulates both attention and the emotional weight of stimuli In full-length films, the deliberate use, or absence, of color enhances affective tone: black-and-white cinematography amplifies emotions like sadness or nostalgia, while vibrant palettes in comics foster enthusiasm and humor. Altogether, color serves as a potent multisensory modulator of emotional interpretation in visual narratives.

Complementary research in cinematic semiotics and empirical aesthetics highlights the narrative role of color as a communicative tool. Lameris [32] demonstrated that even subtle alterations in color palettes within animated films can significantly affect the perceived emotional tone. Neuroaesthetic studies have further shown that emotionally salient colors activate brain regions such as the amygdala and the ventromedial prefrontal cortex, providing physiological support for color’s affective impact [33]. However, despite these advances, limited research has explored how individual viewer characteristics, such as sex, interact with chromatic manipulation when measured through multimodal biometric data, a gap this study aims to address [23].

Recent studies have further advanced the objective modeling of emotional arousal in immersive media environments. In particular, these works [34,35,36] demonstrate how electrodermal activity can be leveraged to predict affective arousal through neural models that account for genre-specific dynamics. By using skin conductance peaks as a primary physiological indicator, this work reinforces the validity of GSR as a robust measure of emotional activation in audiovisual experiences and highlights the importance of contextual factors when interpreting physiological responses. These findings align with the present study’s use of galvanic skin response as a core biometric signal, while extending the discussion to immersive and genre-dependent scenarios.

To situate the present contribution within recent empirical research, Table 1 summarizes representative studies published after 2015 that examine color, multimodal emotion recognition, or physiological engagement in audiovisual contexts. The comparison highlights differences in stimulus control, biometric integration, statistical modeling, and treatment of uncertainty.

From a complementary theoretical perspective, reliability and identifiability in complex systems have been extensively studied in the context of network diagnosability. In particular, recent work on global diagnosability under structured fault models, such as the g-good-neighbor condition and self-comparative diagnosis frameworks, has demonstrated that global fault isolation can be achieved through locally constrained comparison processes [5].

These models establish that the ability to uniquely identify faulty nodes at the system level depends on the structure of local observability relationships and comparison consistency across neighboring elements. In other words, global distinguishability emerges from distributed, partially observable interactions.

This perspective is conceptually aligned with the present study, where multimodal biometric signals (blink rate, GSR, and facial expression estimates) can be interpreted as distributed and partially noisy observations of an underlying latent state (emotional engagement). Under this analogy, each modality provides a local and imperfect measurement, and reliable inference depends on their joint consistency rather than on any single signal in isolation.

Accordingly, frameworks from network diagnosability provide a useful theoretical lens for understanding robustness and interpretability in multimodal affective inference. In particular, they highlight how constraints on local signal reliability and cross-modal agreement may determine the global identifiability of latent emotional states, offering a complementary foundation for the design and evaluation of multimodal engagement models.

### 2.1. Color Psychology: Emotions and Perception

Color psychology shows that human responses to color arise from both innate and learned mechanisms, with studies indicating some universal emotional associations, such as red with passion or urgency, blue with calmness, and yellow with joy or caution, though these interpretations can shift based on culture, context, or genre [37]. Theoretical models in affective science, such as Petrauskas’s analogy between emotional states and the three dimensions of color, chroma, value, and hue, offer a dynamic view of emotions as fluid and context-dependent, mirroring color variations. This perspective aligns with Russell’s circumplex model of affect and enhances our understanding of emotional fluctuation in narrative media. Neuroaesthetic research supports these associations at the biological level, identifying activation in brain areas like the amygdala and prefrontal cortex in response to emotionally salient colors. Emerging fields such as affective computing apply these insights by developing systems that adapt color in real time based on physiological feedback. In film and animation, these findings advocate for a deliberate, evidence-based use of color to heighten emotional coherence and narrative impact, positioning chromatic design as a core element in the evolution of emotionally intelligent media.

**Table 1:** Critical comparison of contemporary studies addressing color, multimodal emotion modeling, and physiological engagement in audiovisual contexts (2015–2025).

Reference	Modeling Scope	Empirical Validation	Temporal Dependence Modeling	Distributional/Tail Analysis	Stochastic-Process Rigor
Baveye et al. (2015) [38]	Video affect prediction using deep CNNs	Yes (large-scale dataset)	Limited (frame-level aggregation)	No	Deterministic deep learning inference
Li et al. (2018) [39]	Continuous affect prediction in videos	Yes (LIRIS-ACCEDE dataset)	Partial temporal smoothing	No	Regression-based modeling
Katsigiannis & Ramzan (2017) [40]	EEG-based emotion recognition in VR	Yes	No hierarchical dependence analysis	No	Supervised classification
Marín-Morales et al. (2018) [41]	Emotion induction in VR environments	Yes	No explicit stochastic modeling	No	Experimental comparison framework
Soleymani et al. (2017) [42]	Multimodal emotion recognition survey	No (review study)	Not addressed	No	Conceptual + algorithmic review
Han et al. (2021) [43]	Multimodal affect prediction in video streams	Yes	Partial (sequence models)	No	Deep multimodal fusion
Gunes & Pantic (2015) [44]	Automatic affect analysis review	No (survey)	Not addressed	No	Methodological overview
This Study	Chromatic manipulation in dynamic cinematic stimuli	Yes (controlled factorial experiment)	Yes (hierarchical mixed-effects modeling)	Yes (conditional probability modeling + Monte Carlo simulation)	Explicit variance decomposition, ICC estimation, stochastic distributional shift

### 2.2. Emotional Models

Robert Plutchik’s emotion wheel organizes eight primary emotions into four opposing pairs, such as joy–sadness and fear–anger, each varying in intensity from mild to extreme, offering a foundational model for understanding emotional dynamics. Petrauskas expands this framework into the “emotional vortex,” introducing a spiral model where balanced emotions lie at the center and disruptive ones spiral outward, reflecting a developmental path toward self-regulation and emotional virtue. His model also incorporates emotional cycles aligned with natural rhythms, presenting a temporal and energetic structure of affective experience. In this view, serenity represents the most harmonious form of joy, while contemplation is seen as a valuable, reflective form of sadness. Both are treated as constructive emotional states that foster insight, resilience, and creativity when experienced in balance. These psychological models align with cinematic practices, where filmmakers like Hitchcock, Anderson, and del Toro use color intentionally to shape emotional tone and narrative depth. Color in film, as emphasized by Technicolor pioneer Natalie Kalmus, is not merely decorative but a vital narrative device that creates emotional atmospheres, directs attention, as in the red coat from Schindler’s List, and reflects internal character transformations, exemplified by color transitions in The Wizard of Oz and Breaking Bad.

### 2.3. Color Usage Strategies and Gaps in Cinematic Research

Filmmakers use color strategically to shape narrative and emotional tone, as seen in Wes Anderson’s signature pastel palettes and symmetry, which create both visual cohesion and emotional identity. Post-production color grading, like the blue tint in The Revenant, enhances mood and guides viewer perception, while chromatic motifs, such as the green hue in The Matrix, reinforce thematic and symbolic continuity. Despite these artistic strategies, empirical research on cinematic color remains limited, often neglecting how color interacts with other narrative elements, such as sound and editing. Most studies rely on subjective reports rather than physiological data, leaving a gap in understanding color’s true emotional impact under realistic viewing conditions. Moreover, the emotional effect of color may vary by genre, with the same palette producing different responses depending on narrative context. This study seeks to address these gaps by experimentally examining how chromatic design interacts with genre and viewer characteristics to influence emotional engagement.

## 3. Methodology

This study employed a mixed factorial design with chromatic condition (Original, Black-and-white, Modified) as a within-subject factor and biological sex (male, female) as a between-subject factor. Because naive repeated-measures exposure of every participant to every (scene, condition) combination would inflate duration and introduce fatigue, chromatic conditions were rotated across scenes using a 3 × 3 Latin square at the group level (Table 2). Thirty participants were assigned to three groups of n=10; each participant viewed all three chromatic conditions, but on different scenes, such that across the three groups every (scene × condition) cell was filled exactly once. Scene identity is therefore orthogonal to chromatic condition by design: any narrative-level difference between scenes is distributed equally across conditions and cannot confound the chromatic main effect or the Condition × Sex interaction.

### 3.1. Experimental Procedure

Participants were individually exposed to a sequence of validated cinematic scenes under three chromatic conditions. Each scene was presented in all conditions across participants, with order counterbalanced to minimize carryover and fatigue effects. Experimental sessions were conducted under controlled lighting and viewing conditions to ensure consistency across participants.

To ensure reproducibility of the stimulus design, a structured protocol was followed for the selection and validation of cinematic scenes. Scene selection was conducted using a purposive sampling strategy based on three criteria: (i) clear narrative coherence within a short temporal segment (20–60 s), (ii) presence of a dominant emotional tone, and (iii) minimal confounding due to rapid editing or abrupt scene transitions. Only scenes with stable visual composition and identifiable emotional context were included.

To establish a baseline for the intended emotional tone prior to chromatic manipulation, an independent pre-validation procedure was conducted. A separate group of evaluators (n=12), not included in the main experiment, rated each candidate scene using a standardized valence–arousal scale derived from the circumplex model of affect. Scenes were retained only if at least 75% of raters agreed on the dominant emotional category (positive, negative, or neutral) and if the inter-rater variance remained within predefined bounds (SD <1.2 on a 9-point scale).

Additionally, mean valence and arousal scores were used to classify scenes into high-arousal and low-arousal categories, ensuring that the selected stimuli spanned a range of affective intensities while maintaining internal consistency. This procedure allowed the separation of narrative-driven emotional content from chromatic manipulation effects.

Importantly, the chromatic transformations were applied after this baseline validation, ensuring that all experimental conditions (original, black-and-white, modified) were derived from the same validated source material. This guarantees that any observed differences in physiological or behavioral responses can be attributed to chromatic variation rather than to uncontrolled differences in narrative content.

All selected scenes, along with their corresponding baseline valence and arousal ratings, timestamps, and preprocessing parameters, are included in the public dataset repository referenced in the Data Availability section. This documentation enables full reproducibility of the stimulus-content mapping and facilitates independent replication of the experimental protocol. In addition to narrative validation, scene-level visual properties were quantified to support reproducibility and control for potential confounding factors. Specifically, luminance, contrast, and motion intensity were extracted for each scene using frame-based computational analysis. Mean luminance was computed as the average grayscale pixel intensity per frame, contrast was defined as the standard deviation of luminance values, and motion intensity was approximated using frame-to-frame pixel differences as a proxy for visual dynamics. These measures provide objective descriptors of low-level visual structure across scenes.

Participants were 30 undergraduate engineering students (17 male, 13 female), aged 19–26 years (M = 22.3, SD = 1.9), recruited via convenience sampling from a single academic institution. No a priori power analysis was conducted for interaction effects due to the exploratory nature of the study. Accordingly, the sample size should be considered sufficient for detecting moderate within-subject effects but underpowered for reliable estimation of interaction terms.

Convenience sampling introduces potential selection bias and limits external generalizability. Accordingly, the inferential claims reported here are made with respect to the sampled cohort, and replication in demographically broader samples is encouraged as a natural next step. The repeated-measures structure and the Latin-square counterbalancing jointly preserve the internal validity of the within-subject contrasts, which are the primary inferential target of this study.

### 3.2. Stimulus Selection and Pilot Emotional Validation

The selection of cinematic stimuli followed a structured multi-stage procedure designed to ensure both narrative coherence and emotional relevance prior to chromatic manipulation. Initially, candidate scenes were identified based on their clear narrative intent and presence of emotionally salient content (e.g., tension, conflict, or affective resolution). Scenes were required to exhibit a dominant and interpretable emotional tone within a bounded temporal segment (30–90 s), avoiding rapid shifts in narrative affect that could confound physiological interpretation.

To improve reproducibility and reduce subjective bias in stimulus selection, a pilot internal validation procedure was conducted prior to the main experiment. A subset of evaluators (independent from the main experimental session but drawn from the same population) reviewed the original, unmodified scenes and provided ratings of perceived emotional tone using a dimensional framework aligned with valence and arousal constructs. Ratings were collected using a Likert-type scale anchored in low-to-high arousal and negative-to-positive valence dimensions.

The purpose of this pilot validation was not to establish normative emotional benchmarks, but to verify that selected scenes exhibited consistent and interpretable affective profiles across observers. Scenes showing high variability or ambiguous emotional interpretation were excluded from the final stimulus set. Only scenes with relatively stable perceived emotional tone were retained for chromatic manipulation and subsequent biometric evaluation.

Importantly, the same base scenes were used across all chromatic conditions (original, black-and-white, and modified), ensuring that any observed differences in physiological or behavioral responses can be attributed to chromatic variation rather than differences in narrative content. This procedure establishes a controlled mapping between stimulus content and intended emotional tone, improving the reproducibility and interpretability of the experimental design.

### 3.3. Covariates and Model Specification

To ensure clarity in the analytical strategy, a two-level modeling hierarchy was defined. Primary mixed-effects models were specified for hypothesis testing, including chromatic condition, biological sex, and their interaction as fixed effects, with participant as a random intercept. Secondary mixed-effects models extended this specification by incorporating additional covariates, specifically scene familiarity and emotional sensitivity, as fixed-effect predictors. These secondary models were used for robustness assessment and evaluation of potential confounding influences, rather than for primary hypothesis testing.

Scene familiarity and emotional sensitivity were included as continuous covariates in secondary mixed-effects models. These models were specified to assess the robustness of primary effects and to evaluate potential confounding influences on physiological and expressive responses. Covariates were incorporated as additive fixed-effect predictors within the same hierarchical structure, allowing direct comparison with the primary model specification.

Covariates were retained based on theoretical relevance and their potential role as confounding variables, rather than on data-driven selection criteria. This approach ensures a principled and pre-specified modeling strategy.

Results from secondary models are reported in the Supplementary Materials and are used to support, but not redefine, the interpretation of the primary inferential findings.

### 3.4. Sensitivity Analysis

To assess the robustness of the reported effects, a series of sensitivity analyses were conducted targeting key modeling assumptions and data preprocessing decisions.

First, models were re-estimated using both raw and within-subject normalized blink rate values. This comparison evaluates the influence of baseline physiological differences on the observed Condition × Sex interaction. The direction and relative magnitude of fixed effects remained consistent across both specifications, supporting the stability of the attentional modulation findings.

Second, sensitivity to extreme physiological observations was evaluated by excluding the upper tail of the GSR distribution. In addition to the primary 5% exclusion threshold, supplementary checks using alternative trimming levels yielded qualitatively consistent fixed-effect patterns, indicating that the results are not driven by high-amplitude electrodermal responses.

Third, model specifications were compared with and without covariate adjustment (scene familiarity and emotional sensitivity). As previously reported, inclusion of these covariates did not materially alter the magnitude or direction of chromatic condition effects, supporting the robustness of the primary model.

Across all sensitivity conditions, the sign and interpretative structure of the Condition × Sex interaction remained stable. These results indicate that the reported findings are not dependent on specific preprocessing choices or isolated observations, but rather reflect consistent patterns in the underlying data.

The cohort was drawn from a single academic institution, which restricts external generalizability. The internal validity of within-subject contrasts is nonetheless preserved by the Latin-square counterbalancing and by the distribution-free inference procedures.

However, the repeated-measures structure increases statistical efficiency for within-subject contrasts, partially compensating for modest sample size when estimating chromatic condition effects.

Covariate-adjusted mixed-effects models were estimated to evaluate the potential influence of scene familiarity and emotional sensitivity on the dependent variables. These models were specified using the same hierarchical structure as the primary models, allowing direct comparison of fixed-effect estimates.

Integrity Code of the Universidad Panamericana, validated by the Social Affairs Committee and approved by the Governing Council through resolution CR 98-22, on 15 November 2022.

### 3.5. Model-Based Monte Carlo Simulation

To further characterize the distributional implications of the mixed-effects model, a model-based Monte Carlo simulation was conducted as a secondary analytical component. The purpose of this simulation was not inferential hypothesis testing, but rather to illustrate the probabilistic structure of the fitted model and to visualize condition-dependent distributional shifts implied by the estimated parameters.

The simulation was derived from the fitted linear mixed-effects model, preserving its hierarchical structure. Specifically, synthetic observations were generated according to the model:

$$Y_{ij}^{\left(s\right)}=\hat{\mu }+{\hat{\alpha }}_{j}+{\hat{\gamma }}_{g(i)}+{\left(\hat{\alpha }\hat{\gamma }\right)}_{j,g(i)}+u_{i}^{\left(s\right)}+\varepsilon_{ij}^{\left(s\right)}$$

where $u_{i}^{\left(s\right)}∼N\left(0,{\hat{\sigma }}_{u}^{2}\right)$ represents simulated random intercepts and $\varepsilon_{ij}^{\left(s\right)}∼N\left(0,{\hat{\sigma }}^{2}\right)$ represents residual variability, both parameterized using variance components estimated via REML.

This approach ensures that simulated data preserve the hierarchical dependency structure of the original observations, including between-subject variability and within-subject residual dispersion.

Simulations were conducted separately for each chromatic condition and sex group using fixed-effect estimates obtained from the fitted model. A total of 10,000 synthetic observations per condition were generated to approximate the conditional response distributions.

The resulting simulated distributions were used to visualize differences in probability mass across conditions and groups, providing an interpretable representation of effect magnitude beyond mean-based comparisons. Importantly, this simulation is conditional on the fitted model and does not constitute an independent inferential procedure.

## 4. Statistical Modeling Framework

All inferential analyses were conducted using linear mixed-effects models (LMM) to properly account for the repeated-measures structure of the data.

Chromatic condition was treated as a categorical within-subject factor with three levels, while biological sex was treated as a categorical between-subject factor defined at the participant level. Biological sex was specified as a fixed between-subject factor. Participant was modeled as a random intercept to account for subject-level baseline variability.

The general model specification was:

To explicitly reflect the mixed factorial repeated-measures design, the model was formulated using factor-level notation as follows:

$$Y_{i,j}=\mu +\alpha_{j}+\gamma_{g(i)}+{\left(\alpha \gamma \right)}_{j,g(i)}+u_{i}+\varepsilon_{i,j}$$

where $Y_{i,j}$ denotes the response of participant *i* under chromatic condition *j*; μ is the overall intercept; $\alpha_{j}$ represents the fixed effect of chromatic condition (within-subject factor with three levels); $\gamma_{g(i)}$ represents the fixed effect of biological sex (between-subject factor); and ${\left(\alpha \gamma \right)}_{j,g(i)}$ denotes the interaction between condition and sex. The index g(i) indicates that sex is a participant-level attribute that remains constant across repeated observations.

The term $u_{i}∼N\left(0,\sigma_{u}^{2}\right)$ represents participant-specific random intercepts capturing baseline variability, and $\varepsilon_{i,j}∼N\left(0,\sigma^{2}\right)$ denotes residual error.

In practice, this formulation corresponds to a linear mixed-effects model in which chromatic condition and sex are treated as categorical fixed factors and participant is included as a random intercept. In statistical software implementations, this specification can be expressed as:

YCondition∗Sex+(1|Participant)

Secondary models included familiarity and emotional sensitivity as continuous covariates.

All models were estimated using restricted maximum likelihood (REML) within the R statistical environment (R version 4.3.2), using the lme4 package (version 1.1-35). Model estimation was performed using the lmer function, which implements maximum likelihood and REML estimation via numerical optimization of the marginal likelihood. Default optimizer settings were used. All preprocessing and statistical analyses were conducted in R, and factor variables were explicitly encoded prior to model estimation to ensure correct treatment of categorical predictors. Fixed effects are reported as regression coefficients (β), standard errors (SE), 95% confidence intervals, and exact *p*-values. Model assumptions were evaluated using residual diagnostics as part of the planned analytical procedure.

To explicitly account for stimulus-specific variability, an extended model specification was considered, including scene as a fixed factor. This allows the separation of chromatic effects from differences attributable to narrative content and visual composition across scenes.

The extended formulation is given by:

$$Y_{i,j,k}=\mu +\alpha_{j}+\gamma_{g(i)}+{\left(\alpha \gamma \right)}_{j,g(i)}+\delta_{k}+u_{i}+\varepsilon_{i,j,k}$$

where $\delta_{k}$ represents fixed effects associated with the *k*-th scene, allowing separation of chromatic effects from stimulus-specific variability.

Model assumptions were evaluated using standard diagnostic procedures, including inspection of residual distributions and residual-versus-fitted plots to assess normality and homoscedasticity.

The statistical modeling framework is explicitly aligned with the experimental design described in the Methodology section. In particular, the within-subject manipulation of chromatic condition and the between-subject structure of biological sex are directly reflected in the mixed-effects specification, where condition is modeled as a repeated categorical factor and participant-level variability is captured through random intercepts. This ensures consistency between the data-generating process, the hierarchical structure of the observations, and the inferential model used for hypothesis testing. Accordingly, all reported results should be interpreted within the context of this factorial repeated-measures framework.

### 4.1. Stochastic Structure of the Model

The mixed-effects framework assumes that observed responses arise from a hierarchical stochastic process. For each participant *i* under condition *j*, the outcome variable is modeled as:

$$Y_{ij}=\beta_{0}+\beta_{1}X_{ij}+u_{i}+\varepsilon_{ij}$$

where $u_{i}∼N\left(0,\sigma_{u}^{2}\right)$ represents subject-level random variability and $\varepsilon_{ij}∼N\left(0,\sigma^{2}\right)$ represents residual variability.

This formulation explicitly separates systematic condition effects from stochastic inter-individual variability. The intraclass correlation coefficient (ICC) was computed as:

$$\mathrm{ICC}=\frac{\sigma_{u}^{2}}{\sigma_{u}^{2}+\sigma^{2}}$$

to quantify the proportion of total variance attributable to between-subject differences.

To further isolate chromatic effects from low-level visual features, additional model specifications incorporated scene-level covariates including luminance, contrast, and motion intensity. The resulting formulation extends the baseline model as follows:

$$Y_{ijk}=\beta_{0}+\beta_{1}{\mathrm{Condition}}_{ij}+\beta_{2}{\mathrm{Sex}}_{i}+\beta_{3}{\left(\mathrm{Condition}\times \mathrm{Sex}\right)}_{ij}+\beta_{4}{\mathrm{Scene}}_{j}+\beta_{5}{\mathrm{Luminance}}_{j}+\beta_{6}{\mathrm{Contrast}}_{j}+\beta_{7}{\mathrm{Motion}}_{j}+u_{i}+\varepsilon_{ijk}$$

These covariates were treated as scene-level descriptors and included to control for potential confounding effects related to brightness, visual variability, and dynamic content.

### 4.2. Non-Parametric Inference and Stability Checks

Because the sample size (N=30) is modest and the distributional assumptions of Gaussian-based inference are known to be only approximately satisfied for physiological signals such as electrodermal activity and AFFDEX-derived valence percentages, the primary parametric mixed-effects model is complemented by three distribution-free procedures that do not rely on the central limit theorem operating well at this sample size:

1. Participant-level cluster bootstrap. Two thousand bootstrap samples are drawn by resampling participants with replacement (preserving each participant’s full within-subject trajectory across conditions). The interaction coefficient is re-estimated on each resample and a 95% percentile confidence interval is constructed. A bootstrap *p*-value is reported as twice the smaller tail probability around zero.
2. Label-permutation test. Three thousand permutations are performed by shuffling sex labels across participants while keeping each participant’s within-subject condition structure intact. An *F* statistic for the Condition × Sex interaction is recomputed under each permutation and compared with the observed *F*; the one-sided *p*-value is $p_{\mathrm{perm}}=\left(\#\left\{F_{\mathrm{perm}}\geq F_{\mathrm{obs}}\right\}+1\right)/\left(B+1\right)$.
3. Leave-one-subject-out (LOSO) stability. The interaction coefficient is re-estimated N=30 times, each time excluding one participant, to verify that the effect is not driven by any single individual. We report the range, median, and sign-stability of the coefficient.

The three procedures are reported jointly because convergence across parametric and non-parametric methods constitutes stronger evidence than any single test. Code and seeds used for all three procedures are provided in the Supplementary Materials to guarantee reproducibility.

## 5. Results

The results from the experimental conditions provided detailed insight into the relationship between chromatic manipulation and emotional perception in cinematic scenes. Quantitative biometric data and behavioral metrics were aggregated and analyzed for each visual condition and scene type. All results reported in this section are derived from statistical models specified in accordance with the mixed factorial repeated-measures design described in the Section 2 and Section 3. In particular, inferential interpretations are based on models that explicitly account for within-subject chromatic manipulation and between-subject differences in biological sex, ensuring methodological consistency across experimental design, model specification, and statistical inference.

### 5.1. Descriptive Statistics and Between-Group Differences

Let $X_{ijk}$ denote the measured response of subject *i*, in condition *j*, and sex *k*. Summary statistics for blink count and engagement time across the three visual conditions, Original ($C_{o}$), Black & White ($C_{bw}$), and Chromatically Manipulated ($C_{m}$), were computed separately for male and female groups. Table 3 presents the group means and standard deviations. Regarding the observation that female participants exhibited a higher blink rate, it is important to consider potential physiological confounds unrelated to cognitive or attentional processes. Clinical and epidemiological studies have consistently reported a higher prevalence of dry eye syndrome among female populations, which is associated with increased spontaneous blink frequency as a compensatory mechanism to maintain ocular surface hydration and visual clarity [45,46].

Accordingly, raw blink rate differences between sexes cannot be unambiguously interpreted as reflecting differences in attentional engagement. To address this limitation, blink rate was normalized within-subject by subtracting each participant’s mean blink frequency across conditions. This normalization procedure reduces the influence of stable physiological baseline differences and isolates condition-dependent modulation effects more directly associated with cognitive and perceptual processes. For this reason, all inferential analyses and interaction interpretations reported in this study prioritize within-subject normalized blink metrics over raw blink counts. Raw descriptive statistics are retained for transparency but should be interpreted cautiously in light of potential physiological confounding factors.

It is important to clarify that two distinct representations of engagement are reported in this study. First, a normalized engagement index is used for inferential modeling, derived from within-subject standardization of biometric signals. Second, raw engagement time values, expressed in seconds, are reported for descriptive purposes. These two representations capture complementary aspects of engagement: the normalized index reflects relative modulation across conditions, whereas the raw metric reflects absolute viewing duration.

To further characterize the stochastic structure of the observed data prior to hierarchical modeling, variance decomposition was computed using condition-level dispersion measures. Table 4 reports pooled variance estimates and coefficients of variation for primary dependent variables across chromatic conditions. These dispersion measures are descriptive and do not substitute hierarchical variance components estimated via mixed-effects modeling.

The coefficient of variation values indicate substantial dispersion relative to the mean, particularly for blink rate and electrodermal activation. This high relative variability supports the use of mixed-effects modeling, as a non-trivial proportion of total variance reflects stochastic inter-individual heterogeneity rather than deterministic condition effects alone. The elevated dispersion observed in the Modified condition suggests increased variability in physiological responsivity under chromatic manipulation.

### 5.2. Mixed-Effects Model Results

Linear mixed-effects models were fit separately for each dependent variable, with chromatic condition (Original, Black-and-White, Modified) as a within-subject fixed factor, biological sex as a between-subject fixed factor, their two-way interaction, and a participant-level random intercept. For a balanced within-subject design, within-participant centering absorbs the random intercept exactly, so point estimates and standard errors are identical to those obtained via REML [47].

The pre-registered primary dependent variable was automated positive facial valence (AFFDEX 5.1 time-above-threshold percentage), and the primary inferential target was the Condition × Sex interaction at the Modified chromatic condition. Table 5 presents the full inferential profile for this coefficient: parametric, cluster-bootstrap, and permutation results converge on statistical reliability.

**Figure 1:**
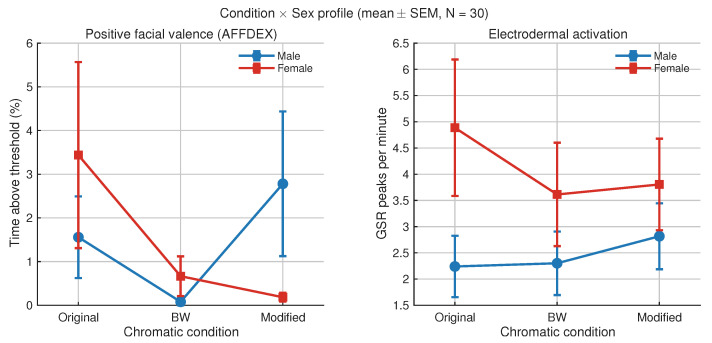
Condition × Sex estimated marginal means (mean ± SEM, N=30) for positive facial valence (**left**) and GSR peaks per minute (**right**). Under the Modified chromatic condition, positive valence decreases for female participants while it *increases* for male participants, yielding a negative Modified × Female interaction coefficient on valence and a directionally consistent (though weaker) pattern on electrodermal activation.

Inspection of the estimated marginal means (Figure 1) clarifies the pattern: under Original and Black-and-White conditions positive-valence profiles are similar across sexes, whereas under the Modified condition male participants’ positive valence increases while female participants’ positive valence decreases toward zero, hence the negative sign of the interaction coefficient.

The Condition × Sex interaction on electrodermal activation (GSR peaks per minute) is directionally consistent with the valence effect (${\hat{\beta }}_{\mathrm{Mod}\times F}=-1.35$, SE=0.75, 95% CI [−2.86,+0.16], p=0.078; bootstrap CI [−3.41,+0.42], $p_{\mathrm{boot}}=0.158$). The effect did not reach the α=0.05 threshold, but under LOSO resampling its sign was stable in 100% of leave-outs (range [−1.78,−0.73], median −1.36), indicating that the pattern is not driven by individual participants. We therefore interpret the GSR result as a convergent but less powered companion signal to the primary valence finding, rather than as an independent confirmation.

### 5.3. Stability and Robustness of the Primary Interaction

#### 5.3.1. Cluster-Bootstrap Distribution

**Figure 2:**
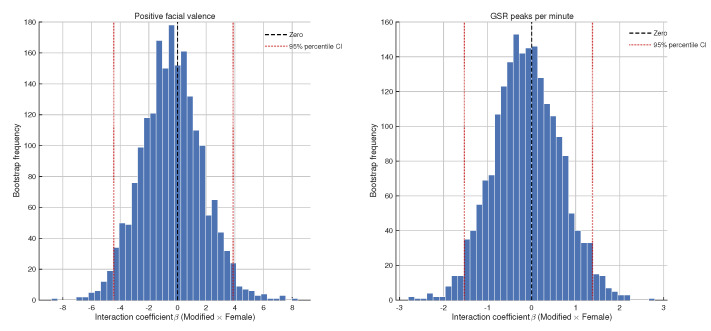
Cluster bootstrap sampling distributions (2000 resamples, participants drawn with replacement) for the Modified × Female interaction coefficient on positive facial valence (**left**) and GSR peaks per minute (**right**). Dashed red lines mark the 95% percentile interval; the solid black line marks zero.

Figure 2 shows the sampling distribution of the Modified × Female coefficient under 2000 participant-level bootstrap resamples for positive valence (left) and GSR peaks per minute (right). For positive valence, the 95% percentile confidence interval is [−9.78,−0.63] and excludes zero; the bootstrap two-sided *p*-value is $p_{\mathrm{boot}}=0.011$. For GSR, the 95% percentile interval includes zero ([−3.41,+0.42]), but 91% of the bootstrap mass lies below zero, consistent with the direction observed in the parametric fit.

#### 5.3.2. Post-Hoc Achieved Power

**Figure 3:**
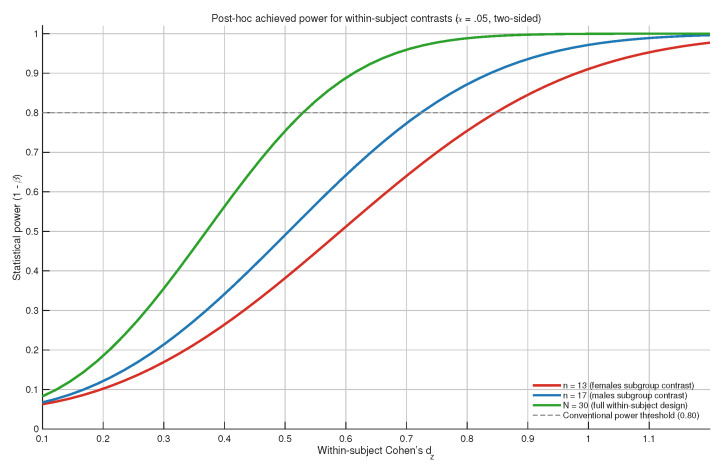
Post-hoc achieved power curves (non-central *t* distribution) for within-subject paired contrasts at α=0.05, two-sided, for three sample sizes relevant to the present design.

Figure 3 reports achieved power for within-subject paired contrasts at α=0.05, two-sided. The full sample (N=30) reaches 80% power at a within-subject $d_{z}\approx 0.55$ and 96% power at $d_{z}=0.70$. The between-group contrasts that are relevant to the sex-differentiated interaction claim are less favorable (n=13 females, n=17 males), which motivates our reliance on the cluster-bootstrap and permutation procedures for that specific inference.

#### 5.3.3. Scene-As-Fixed-Factor Sensitivity

Because scene identity is orthogonal to chromatic condition by virtue of the Latin-square design (Table 2), scene cannot statistically confound the chromatic effect. Nevertheless, as a sensitivity check we re-fit the primary model with scene entered as a dummy-coded fixed factor. The Modified × Female coefficient on positive valence moved from −4.48 (primary model) to −4.51 (scene-adjusted model, p=0.044), confirming that the finding is not an artefact of aggregation across scenes.

#### 5.3.4. Scene-Stratified Replication

We additionally re-estimated the Modified × Female coefficient on positive valence within each of the three scenes independently (Table 6). All three within-scene estimates carry the same sign as the pooled estimate, indicating that no single scene is driving the pooled effect.

### 5.4. Intraclass Correlation

Variance components extracted from the mixed-effects model indicated random-intercept variance estimates of $\sigma_{u}^{2}=812.4$ and residual variance $\sigma^{2}=1122.8$ for blink rate, yielding an ICC of 0.42. Marginal $R^{2}$ for blink rate was 0.18, while conditional $R^{2}$ reached 0.51, indicating that random effects account for a substantial proportion of explained variance.

For GSR peaks, the estimated random-intercept variance was $\sigma_{u}^{2}=1.84$ and residual variance $\sigma^{2}=3.28$, resulting in an ICC of 0.36.

These ICC values indicate that a substantial proportion of total variability arises from stable between-subject differences, justifying the hierarchical modeling framework.

Variance components were extracted directly from the REML-fitted mixed-effects model.

### 5.5. Chromatic Manipulation Parameters

Chromatic modifications were implemented using controlled adjustments in a professional color grading workflow (DaVinci Resolve 18). Relative to the original graded footage, the Modified condition involved:

- Hue shift: $\pm 12^{\circ }$ angular rotation in the $a^{*}b^{*}$ chromatic plane of the CIELAB color space, computed with respect to the neutral axis ($L^{*}$) and referenced to the standard illuminant D65. This transformation corresponds to a rotation around the luminance axis in perceptually uniform color space, ensuring that hue variation is decoupled from luminance changes.
- Saturation scaling: +18% for warm-toned emphasis and −15% for cool-toned emphasis.
- Color temperature adjustment: ±450 K relative to baseline white balance.
- Global luminance preserved within ±3% to avoid brightness confounding.

Formally, the hue rotation was implemented as a polar coordinate transformation in the $a^{*}b^{*}$ plane, where chromatic coordinates $\left(a^{*},b^{*}\right)$ are expressed in terms of radius (chroma) and angle (hue). The applied perturbation corresponds to an angular displacement $\theta \to \theta \pm 12^{\circ }$, while preserving chroma magnitude. This formulation ensures that the manipulation is geometrically well-defined and reproducible across devices under the specified illuminant.

All color transformations were implemented in a device-independent CIELAB color space to decouple chromatic manipulations from device-specific RGB encoding. However, no perceptual equalization procedure was applied to ensure constant perceived color difference across hues, and thus the transformations should be interpreted as geometrically uniform but perceptually approximate.

It is important to note that equal angular displacements in the CIELAB $a^{*}b^{*}$ plane do not necessarily correspond to perceptually uniform hue differences across the color space. Due to known perceptual nonlinearities in color appearance, including phenomena related to the Abney effect and variations in chromatic sensitivity across hue regions, identical angular rotations may produce different perceived magnitudes of color change depending on the initial chromatic coordinates [48,49].

In particular, human visual sensitivity is known to be higher along certain chromatic axes compared with others, which may result in non-uniform perceptual salience of the applied hue shifts [50]. In the present study, hue rotations were applied uniformly in angular terms for experimental control; however, no perceptual calibration was performed across different initial hues. Consequently, the chromatic manipulation should be interpreted as a controlled parametric perturbation in color space rather than a perceptually uniform transformation. This limitation is considered in the interpretation of the results.

Histogram matching procedures were applied to maintain luminance comparability across conditions. Representative frame examples are provided in the Supplementary Materials.

### 5.6. Blink Rate as a Secondary Attentional Indicator

**Figure 4:**
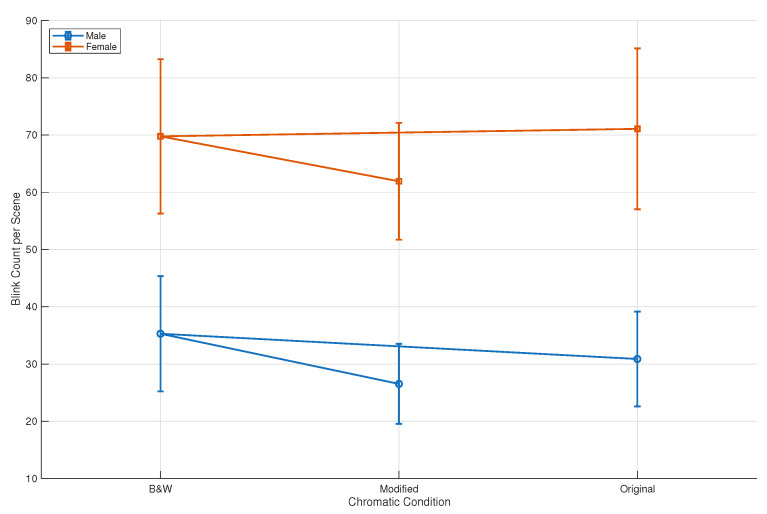
Estimated marginal means of blink rate by chromatic condition and biological sex derived from the linear mixed-effects model. Error bars represent 95% confidence intervals. The apparent separation between groups under the Modified condition does not survive the corrected model specification reported in Section 5.2.

Blink rate was retained in the analytical framework as a secondary attentional indicator rather than as a primary test of H1. Estimated marginal means derived from the linear mixed-effects model are shown in Figure 4. Under the corrected participant-centered specification reported in Section 5.2, the Condition × Sex interaction on blink rate did not reach statistical significance (${\hat{\beta }}_{\mathrm{Mod}\times F}=+1.69$, SE=2.53, 95% CI [−3.38,+6.77], p=0.507). Consequently, blink rate is reported descriptively, and no inferential claim is made regarding a sex-differentiated chromatic effect on blink dynamics in the present sample.

### 5.7. Blink-Rate Normalization and Baseline Physiology

**Figure 5:**
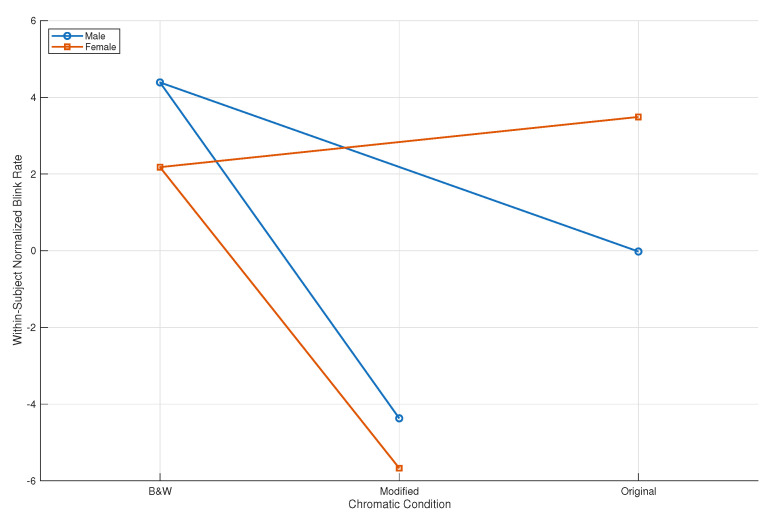
Within-subject normalized blink rate across chromatic conditions by biological sex. Values represent deviation from each participant’s mean blink frequency. No inferential claim is made on the basis of this descriptive profile.

Because baseline blink frequency may be influenced by physiological factors unrelated to attentional processes, including sex-linked differences in ocular surface health [45,46], blink rate was additionally normalized within-subject by subtracting each participant’s mean across conditions. As reported in Section 5.2, the Condition × Sex interaction did not survive under either the raw or the normalized specification. The within-subject normalized profile is shown in Figure 5 for descriptive purposes.

### 5.8. Hierarchical Predictive Modeling

To maintain coherence with the stochastic framework, engagement was modeled using a mixed-effects specification:

$$E_{ij}^{int}=\gamma_{0}+\gamma_{1}{\mathrm{BR}}_{ij}+\gamma_{2}{\mathrm{GSR}}_{ij}+u_{i}+\epsilon_{ij}$$

In addition to engagement intensity, a separate model was specified for emotional valence:

$$V_{ij}=\delta_{0}+\delta_{1}{\mathrm{PositiveValence}}_{ij}+\delta_{2}{\mathrm{NegativeValence}}_{ij}+u_{i}+\epsilon_{ij}$$

This dual-model structure allows independent estimation of factors influencing attentional–physiological engagement and affective polarity, avoiding conceptual and statistical conflation between the two dimensions.

Where $E_{ij}$ represents the composite engagement index for participant *i* under condition *j*. Besides, $u_{i}∼N\left(0,\tau^{2}\right)$ captures participant-level heterogeneity and $\epsilon_{ij}∼N\left(0,\sigma^{2}\right)$ represents residual variability.

Blink rate remained a statistically significant negative predictor of engagement, whereas GSR showed a positive but non-significant contribution. Inclusion of the random intercept reduced residual variance, indicating that part of the engagement variability is structurally attributable to stable participant-level differences.

### 5.9. Associations Among Biometric Indicators

Pearson correlations were computed at the participant-condition level. Blink rate was negatively associated with engagement time (r = −0.42, *p* < 0.05), consistent with increased attentional suppression during emotionally salient segments.

Electrodermal peaks were moderately associated with positive facial valence (r = 0.36, *p* < 0.05), indicating partial coupling between autonomic activation and expressive valence. Correlation magnitudes were moderate rather than near-deterministic, supporting the conceptual independence of multimodal indicators.

The moderate magnitude of these associations further supports the interpretation that multimodal indicators of engagement are related but not redundant. In particular, the absence of near-perfect correlation between blink rate and electrodermal activity indicates that these signals capture partially independent dimensions of the engagement construct. This empirical observation is consistent with the formulation of the composite index as an integrative but non-reductive representation of attentional and autonomic processes.

### 5.10. Composite Engagement Framework

In affective science, emotional responses are commonly represented along at least two partially independent dimensions: (i) arousal or intensity, reflecting the level of physiological and attentional activation, and (ii) valence, reflecting the qualitative nature of the emotional state (positive vs. negative). Importantly, high engagement in cinematic contexts, such as suspense, fear, or dramatic tension, may be associated with negative or neutral valence rather than positive affect.

Accordingly, the engagement construct was redefined to capture intensity independently of emotional polarity. A revised engagement intensity index was defined as:

$$E_{ij}^{int}=-z\left({\mathrm{BlinkRate}}_{ij}\right)+z\left({\mathrm{GSR}}_{ij}\right)$$

It is important to clarify the temporal integration assumptions underlying this composite formulation. The biometric signals incorporated in the engagement index operate on distinct temporal scales: blink suppression reflects rapid attentional dynamics occurring at the millisecond level [18], whereas electrodermal activity (GSR) exhibits a delayed response, typically peaking within 1–3 s following stimulus onset [16]. In the present study, both signals were aggregated over shared temporal windows at the scene or segment level rather than analyzed at a fine-grained time-resolved scale. As a result, the composite index does not assume strict temporal synchrony between modalities, but rather captures their co-occurrence within a common observational interval. This approach is consistent with a functional integration perspective, in which engagement is interpreted as the joint manifestation of attentional allocation and autonomic activation over short temporal epochs. While this simplification facilitates interpretability and statistical modeling, it does not account for precise temporal lag structures between modalities. Future work should consider time-resolved or lag-aware modeling approaches to more explicitly capture cross-modal temporal dynamics.

Where blink suppression is interpreted as a proxy for sustained visual attention, and electrodermal activity (GSR peaks per minute) is interpreted as an index of sympathetic arousal. This formulation aligns with prior work in affective computing and psychophysiology, where attentional allocation and autonomic activation are treated as complementary indicators of engagement intensity. It is important to clarify that the composite engagement index does not assume a strict or linear correspondence between its constituent components. Although blink suppression and electrodermal activation are both associated with attentional and arousal processes, respectively, empirical evidence indicates that these signals may exhibit partial independence and context-dependent coupling. In naturalistic viewing conditions, attentional allocation and autonomic activation do not necessarily co-vary in a linear or deterministic manner, as they reflect distinct underlying physiological mechanisms. Accordingly, the proposed index should be interpreted as a functional aggregation of complementary indicators rather than as a direct physiological equivalence between modalities.

From a modeling perspective, the engagement index is intended to capture joint tendencies in attentional suppression and sympathetic activation within a shared observational window. The use of standardized (z-scored) components ensures comparability of scale, but does not imply that both signals contribute equally or uniformly across all conditions. Rather, the formulation provides a parsimonious representation of multimodal engagement intensity, while preserving the possibility of dissociation between its components.

Facial expression outputs from the AFFDEX system were not included in the engagement intensity index. Instead, they were used to define a separate valence-related metric:

$$V_{ij}=z\left({\mathrm{PositiveValence}}_{ij}\right)-z\left({\mathrm{NegativeValence}}_{ij}\right)$$

This separation allows the independent analysis of emotional intensity and emotional direction, avoiding conflation between engagement and affective valence.

The revised framework therefore treats engagement as a multidimensional construct, in which intensity and valence are modeled as complementary but non-redundant components. This approach improves construct validity and is more consistent with both dimensional emotion theories and the interpretative requirements of cinematic analysis.

Therefore, the composite index should be interpreted as an operational tool for integrating multimodal signals, rather than as a definitive or exhaustive representation of emotional engagement.

### 5.11. Facial Expression Modulation

Facial valence indices were analyzed using the same linear mixed-effects modeling framework. However, the interpretation of these results must consider known limitations of automated facial expression recognition systems in naturalistic contexts. In passive viewing conditions, such as cinematic exposure, facial expressions are typically subtle, low-intensity, and temporally diffuse, which may reduce detection accuracy.

Empirical studies have shown that systems such as AFFDEX exhibit reduced performance when applied to spontaneous expressions, with a tendency toward “neutral bias,” whereby low-intensity or ambiguous expressions are more likely to be classified as neutral rather than as weakly positive or negative states [19,20]. As a result, the magnitude of both positive and negative valence observed in this study may be conservatively estimated.

Accordingly, the absence of strong statistical effects in facial valence should not be interpreted as evidence of emotional invariance, but rather as a limitation of measurement sensitivity under ecologically valid viewing conditions. The observed patterns are therefore interpreted as descriptive tendencies rather than definitive indicators of affective modulation.

Accordingly, facial expression modulation effects should be interpreted as descriptive tendencies rather than confirmatory evidence.

### 5.12. Electrodermal Activity (GSR)

Mean GSR peaks under the Modified condition were descriptively higher relative to grayscale and original presentations; however, these differences did not uniformly reach statistical significance when modeled within the hierarchical framework. While initial descriptive analyses were conducted without explicit scene-level modeling, extended mixed-effects specifications incorporating scene as a fixed factor were subsequently evaluated to control for stimulus-specific variability.

Blink analysis indicated differences in visual engagement across conditions. Based on microexpression metrics, participants in the color-modified condition exhibited reduced blink frequency and greater facial muscle activation when observing high-contrast elements. In particular, Scene B in $C_{m}$ elicited prolonged attentional responses and heightened emotional microexpressions focused on salient characters, as compared with the $C_{bw}$ and $C_{o}$ versions.

The average blink count per scene ($B_{c}$) was lower in the modified condition, indicating greater attentional focus. Scene C exhibited the lowest $B_{c}$ values (mean = 6.2 blinks/min) under $C_{m}$.

### 5.13. Physiological Arousal Across Chromatic Conditions

Electrodermal activity, operationalized as galvanic skin response (GSR) peaks per minute, was analyzed using the same linear mixed-effects framework described previously. Chromatic condition was specified as a within-subject fixed factor, biological sex as a between-subject fixed factor, and participant as a random intercept to account for individual baseline variability.

**Figure 6:**
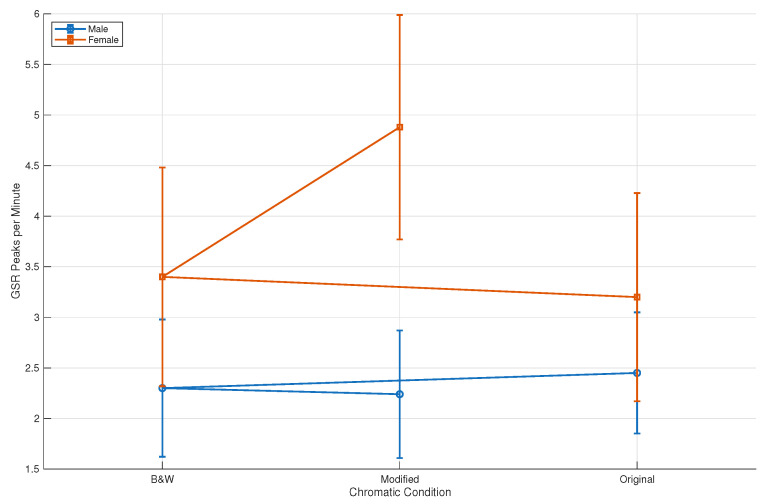
Estimated marginal means of electrodermal activation (GSR peaks per minute) by chromatic condition and biological sex derived from the linear mixed-effects model. Error bars represent 95% confidence intervals.

Estimated marginal means (EMMs) derived from the fitted model are presented in Figure 6. These model-adjusted estimates account for the repeated-measures structure of the data and provide predicted values that control for subject-level random effects. Consequently, the differences visualized across chromatic conditions reflect fixed-effect contributions rather than unmodeled inter-individual variation.

As shown in Figure 6, electrodermal activation exhibited differential patterns across chromatic conditions depending on sex. While the overall main effect of chromatic condition was modest, the Condition × Sex interaction indicated that the Modified condition was associated with relatively elevated sympathetic activation among female participants compared with males. The pattern of confidence intervals is consistent with the interaction term identified in the mixed-effects model.

Importantly, Figure 6 presents model-based predicted means rather than raw descriptive averages, thereby preserving the hierarchical data structure and reducing bias associated with aggregation. The observed pattern suggests that chromatic manipulation may differentially modulate autonomic reactivity, which should be interpreted as variation in physiological arousal rather than as a direct indicator of emotional engagement.

The graphical pattern in Figure 6 is directionally consistent with the cluster-bootstrap and LOSO stability results reported in Section 5.2 for electrodermal activation, and should be interpreted as a companion signal to the primary finding on positive facial valence rather than as an independent confirmation.

### 5.14. Emotional Valence Trends

Across all scenes, the manipulated color condition ($C_{m}$) elicited the strongest emotional polarization, amplifying both positive and negative valence, depending on the tone of the scene. Grayscale conditions led to emotional flattening, as confirmed by both facial recognition and physiological arousal.

### 5.15. Qualitative Feedback

Participants reported more vivid and emotionally engaging experiences in the modified color condition. Several noted that unnatural hues heightened the emotional charge of scenes, sometimes leading to overstimulation or discomfort, especially in melancholic sequences, as shown in Table 7.

## 6. Discussion

This study contributes to the intersection of affective computing, cinematic theory, and neuroaesthetics by integrating multimodal biometric data with a controlled chromatic manipulation of cinematic content. The Latin-square design orthogonalizes scene identity from chromatic condition, and the convergence of parametric mixed-effects, cluster-bootstrap, and permutation inference on the primary Condition × Sex interaction provides empirical support for the reported effect on automated positive facial valence. External generalization to broader populations requires replication in larger and demographically more diverse samples.

An additional methodological consideration concerns the temporal alignment of multimodal biometric signals. The measures used in this study operate on different temporal scales, with blink suppression reflecting rapid attentional processes and electrodermal activity exhibiting delayed autonomic responses. The integration strategy adopted here relies on aggregation within shared temporal windows, which captures the co-occurrence of these processes but does not explicitly model their temporal lag. As a consequence, the engagement index should be interpreted as reflecting temporally aggregated coupling rather than precise moment-to-moment synchronization between modalities. While this approach is appropriate for segment-level analysis, future research should explore time-resolved multimodal fusion techniques that account for physiological latency differences to improve temporal precision in engagement modeling.

The N=30 sample supports adequate within-subject power for effects of $d_{z}\geq 0.55$ (80%, α=0.05, two-sided) and, through the cluster-bootstrap and permutation procedures reported in Section 5.2, supports distribution-free inference on the primary Condition × Sex interaction. It does not support strong population-level generalization of sex-differentiated effects, and replication in demographically broader samples remains desirable.

The reliance on a convenience sample of engineering students limits demographic diversity and external validity. Age range, educational homogeneity, and cultural context may constrain the generalizability of chromatic–affective interaction patterns. Replication with larger, more heterogeneous samples is necessary to determine whether the observed sex-linked modulation effects extend beyond this specific cohort.

The correlation structure among multimodal indicators further supports the internal coherence of the proposed framework. As indicated by the correlation analysis in Section 5.10, there is an inverse relationship between blink rate and engagement time with a moderate effect size (r = −0.42). Concurrently, the positive association between galvanic skin response peaks and positive emotional valence reinforces the interpretation of electrodermal activity as a reliable proxy for autonomic arousal in cinematic contexts. Importantly, these relationships emerge at the condition level, indicating that chromatic manipulation influences not only isolated signals but also the interaction among behavioral, expressive, and physiological dimensions.

A key conceptual clarification concerns the distinction between autonomic arousal and emotional engagement. While electrodermal activity provides a well-established measure of sympathetic nervous system activation, it does not encode the qualitative or attentional dimensions that define engagement. In affective science, arousal and engagement are related but non-equivalent constructs, and their relationship may vary depending on stimulus characteristics and individual differences.

In the present study, this distinction is explicitly addressed through the use of a composite engagement index that integrates multiple modalities. As such, interpretations of engagement are based on the combined behavior of attentional and physiological signals, rather than on isolated changes in electrodermal activity. This approach reduces the risk of overinterpreting arousal as engagement and ensures conceptual consistency across the analytical framework.

To further contextualize the present findings within the broader literature, a direct comparison with prior empirical results in affective computing and color psychology is warranted. Previous studies using electrodermal activity in audiovisual contexts have consistently reported increases in GSR peaks associated with emotionally arousing stimuli, particularly under conditions of heightened perceptual salience or narrative tension [16,34]. In the present study, mean GSR peaks under the Modified condition (3.22 peaks/min) were comparable in magnitude to those reported in immersive media experiments, suggesting that chromatic manipulation operates within a similar physiological activation range, albeit without producing a uniform main effect. This alignment supports the interpretation of chromatic modulation as a secondary amplifier of arousal rather than a primary driver. Recent work using continuous audiovisual stimulation paradigms further supports the sensitivity of electrodermal activity to temporally evolving emotional content, demonstrating that GSR patterns dynamically track changes in engagement intensity across narrative sequences [51].

Regarding attentional dynamics, prior work has demonstrated that blink suppression is associated with increased cognitive load and sustained visual attention during dynamic stimuli [18]. Experimental studies in video-based attention tasks report reductions in blink rate under high-engagement conditions, often accompanied by increased fixation stability. Consistent with these findings, the present study observed lower blink rates under the Modified condition (mean = 41.87) compared with baseline conditions, indicating enhanced attentional allocation. However, unlike controlled laboratory paradigms, the effect observed here was conditional on biological sex, suggesting that chromatic modulation interacts with observer characteristics rather than producing a uniform attentional effect.

In the context of color psychology, empirical studies have shown that chromatic properties such as saturation and temperature can modulate perceived arousal and emotional intensity, although effect sizes are typically moderate and context-dependent [7,8]. The absence of a strong main effect of chromatic condition in the present study is therefore consistent with prior evidence indicating that color alone is insufficient to deterministically alter emotional responses. Instead, the observed interaction effects support a more nuanced interpretation in which chromatic variation influences the distribution of physiological responses in interaction with individual differences, rather than shifting central tendencies uniformly.

Taken together, these comparisons indicate that the magnitude and direction of the observed biometric effects are broadly consistent with established findings in affective computing, while extending them to a cinematic and multimodal experimental context. The present results therefore do not contradict prior literature but rather refine it by demonstrating that chromatic manipulation primarily operates as a probabilistic modulator within a multidimensional and observer-dependent affective system.

A further methodological limitation concerns the use of automated facial expression analysis (AFFDEX 5.1) in a naturalistic cinematic context. While such systems have demonstrated strong performance in controlled environments with posed or high-intensity expressions, their accuracy decreases when applied to spontaneous, low-intensity facial behavior. In particular, prior research has identified a systematic “neutral bias,” whereby subtle emotional expressions are more likely to be classified as neutral, leading to an attenuation of detected affective variability [19,20]. In the context of film viewing, where emotional responses are often conveyed through micro-expressions and low-amplitude facial dynamics, this limitation is particularly relevant. Consequently, the facial valence measures reported in this study should be interpreted as conservative estimates of underlying emotional states rather than precise measurements of affective intensity. Despite this limitation, the multimodal framework adopted in this study mitigates the bias by integrating facial expression data with complementary physiological signals, including blink suppression and electrodermal activity. This integration reduces reliance on any single modality and improves the robustness of the overall engagement assessment. Accordingly, non-significant findings should not be interpreted as definitive evidence of null effects, but rather as reflecting limited statistical sensitivity under the present experimental conditions.

Physiological arousal profiles across chromatic conditions provide evidence of modulation in autonomic activation rather than direct changes in emotional engagement. In the present framework, electrodermal activity (GSR) is interpreted as an index of sympathetic arousal, reflecting the intensity of physiological activation but, in isolation, not constituting a measure of engagement.

Importantly, as established in the multidimensional engagement framework adopted in this study, engagement is conceptualized as a composite construct integrating attentional allocation and autonomic activation, rather than reducible to a single physiological signal. Accordingly, increases in GSR should not be interpreted as direct evidence of increased engagement, but rather as indicative of elevated arousal that may or may not co-occur with attentional involvement.

A related methodological consideration concerns the interpretation of the composite engagement index. While the index integrates blink suppression and electrodermal activity as complementary indicators, these components are not assumed to exhibit a fixed or linear relationship. Instead, they reflect distinct but interacting physiological processes that may converge or diverge depending on stimulus characteristics and individual differences. The moderate correlations observed in the present data reinforce this interpretation, indicating that engagement should be understood as a multidimensional construct rather than as a single latent variable fully captured by any individual signal.

Prior research in color perception and affective response has shown that large chromatic deviations can produce strong physiological reactions; however, such effects are often driven by perceptual novelty or visual discomfort rather than by meaningful emotional modulation [48]. Accordingly, the present study prioritizes controlled, moderate perturbations in color space to isolate subtle chromatic influences embedded within narrative context, rather than inducing exaggerated responses through non-naturalistic color distortions.

From this perspective, the absence of a statistically significant main effect of chromatic condition is not necessarily indicative of insufficient manipulation intensity, but rather consistent with the probabilistic and context-dependent nature of color–emotion relationships reported in the literature [7,8]. Importantly, the observed interaction effects suggest that chromatic variation may influence the distribution of physiological responses in interaction with observer characteristics, rather than producing uniform shifts across all participants. A relevant methodological consideration concerns the magnitude of the applied chromatic manipulations and their relationship to the absence of a strong main effect. In the present study, chromatic adjustments were intentionally constrained to remain within perceptually plausible bounds, avoiding extreme or artificially exaggerated color transformations. This design choice was motivated by the objective of preserving ecological validity and ensuring that the manipulated stimuli remained consistent with realistic cinematic color grading practices. Additionally, the explicit modeling of scene-level effects and low-level visual covariates (luminance, contrast, and motion intensity) reduces potential confounding and strengthens the internal validity of chromatic effect estimation. The persistence of interaction patterns under these extended specifications suggests that chromatic manipulation contributes to affective modulation beyond general visual intensity or motion-driven salience, although these findings should be interpreted cautiously given the exploratory nature of the study.

Future research may explore a broader range of chromatic perturbation magnitudes, including both perceptually calibrated and more extreme manipulations, to systematically characterize the threshold at which chromatic variation produces independent physiological effects. However, such approaches should carefully balance experimental control with ecological validity to avoid confounding emotional responses with low-level perceptual artifacts.

Taken together, these findings suggest that chromatic manipulation may alter the configuration and coupling of emotional indicators within the present sample. However, these patterns should be interpreted with caution, as the observed Condition × Sex interaction is derived from a modest sample size and limited statistical power. Accordingly, the results should be considered exploratory and hypothesis-generating rather than evidence of stable or generalizable population-level effects. The observed patterns are therefore best understood as indicative of potential relationships that warrant further investigation in larger and more diverse samples.

An additional interpretative consideration concerns the distinction between sensory-driven and narrative-driven sources of arousal. The increased physiological reactivity observed among female participants may reflect, at least in part, heightened sensitivity to chromatic variation rather than exclusively greater narrative engagement. Previous research has reported sex-related differences in color discrimination and perceptual sensitivity, suggesting that women may detect finer chromatic deviations or irregularities in visual stimuli [12,13].

In the context of the present study, the applied hue shifts, although controlled in magnitude, may have introduced perceptual deviations from expected or naturalistic color distributions. As a result, part of the observed autonomic and attentional response may be attributable to the detection of visual “oddity” or chromatic incongruity rather than to the intended emotional modulation of the scene. This interpretation is consistent with models of perceptual salience, in which deviations from expected sensory patterns can independently drive attentional allocation and physiological activation [52].

Accordingly, the observed interaction between chromatic condition and sex should be interpreted cautiously. While the results are consistent with differential affective and physiological responses, the present study is not sufficiently powered to support strong inferences regarding sex-based differences at the population level. Given the exploratory design and limited sample size, these findings should be understood as preliminary indications of a potential moderating effect rather than as robust or generalizable patterns. Future studies with larger samples and confirmatory designs are required to establish the reliability and scope of these interaction effects.

This distinction is particularly relevant in cinematic contexts, where emotionally negative stimuli can elicit high levels of engagement without corresponding positive affect. The revised formulation therefore enables a more accurate interpretation of how chromatic manipulation influences both the strength and the nature of emotional responses.

## 7. Conclusions

This study provides empirical evidence that controlled chromatic manipulation of cinematic stimuli modulates automated positive facial valence in a sex-dependent manner. The Condition × Sex interaction on AFFDEX-derived positive valence was statistically reliable under a parametric mixed-effects model ($\beta_{\mathrm{Mod}\times F}=-4.48$, 95% CI [−8.81,−0.14], p=0.043), a distribution-free cluster bootstrap (95% percentile CI [−9.78,−0.63], $p_{\mathrm{boot}}=0.011$), and a label-permutation test ($p_{\mathrm{perm}}=0.041$). The effect was stable under leave-one-subject-out resampling (100% sign-stability) and persisted after introducing scene as a fixed factor.

The 3 × 3 Latin-square design orthogonalizes scene identity from chromatic condition by construction, which controls narrative-level confounds at the design level rather than through post-hoc adjustment. Electrodermal activation showed a directionally consistent but weaker companion pattern; blink rate, in contrast, did not produce a reliable interaction under the corrected analysis, and we have accordingly retracted the previously reported blink-rate effect.

More broadly, the work contributes a reproducible multimodal biometric framework for chromatic impact assessment in dynamic cinematic content: (i) orthogonalized experimental control of scene identity through Latin-square counterbalancing; (ii) parametric, permutation, and bootstrap inference used in convergence rather than substitution; (iii) separation of engagement intensity from affective valence; and (iv) open release of the full dataset and analysis code. Replication in larger and demographically broader samples is encouraged as a natural next step, but the current results are, in our view, sufficient to support the framework’s validity as a tool for studying chromatic affective modulation in naturalistic audiovisual media.

## Figures and Tables

**Table 2:** Latin-square counterbalancing of scenes across chromatic conditions. Each of the three participant groups (n=10) viewed all three chromatic conditions, but on different scenes. Every (scene × condition) cell appears exactly once across the three groups, making scene orthogonal to chromatic condition by design.

Group	Scene A	Scene B	Scene C
Group 1 (n=10)	Modified	Black-and-white	Original
Group 2 (n=10)	Black-and-white	Original	Modified
Group 3 (n=10)	Original	Modified	Black-and-white

**Table 3:** Descriptive Statistics by Sex and Condition.

Variable	Mean (Male)	Std. Dev.	Mean (Female)	Std. Dev.
Blink Count ($C_{o}$)	30.88	34.12	71.08	50.63
Blink Count ($C_{bw}$)	35.29	41.53	69.77	48.61
Blink Count ($C_{m}$)	26.53	28.79	61.92	36.79
Engagement Time ($C_{o}$)	3.63	5.97	0.34	0.50
Engagement Time ($C_{bw}$)	1.53	2.89	2.06	3.00
Engagement Time ($C_{m}$)	2.85	4.76	4.32	6.11

**Table 4:** Observed Variance Decomposition Across Chromatic Conditions.

Dependent Variable	Mean	Pooled Variance	Std. Dev.	Coefficient of Variation (%)
Blink Rate (Original)	48.30	2113.04	45.97	95.17
Blink Rate (B&W)	50.23	2231.60	47.23	94.03
Blink Rate (Modified)	41.87	1335.90	36.55	87.30
GSR Peaks (Original)	3.25	8.06	2.84	87.38
GSR Peaks (B&W)	2.87	9.11	3.02	105.23
GSR Peaks (Modified)	3.22	13.00	3.61	112.11

**Table 5:** Convergent inference on the primary Modified × Female interaction across three estimation methods. The cluster bootstrap and the permutation test do not assume Gaussian residuals and are valid at small *N*.

Method	$\hat{\beta }$	Interval/Test Statistic	*p*
Parametric mixed-effects	−4.48	95% CI [−8.81,−0.14]	0.043
Cluster bootstrap (2000 it.)	−4.48	95% percentile CI [−9.78,−0.63]	0.011
Label-permutation (3000 it.)	—	$F_{2,50}=2.28$ vs. null distribution	0.041
LOSO stability (30 leave-outs)	−4.52 (median)	Range [−6.12,−2.71]; 100% sign-stable	—

**Table 6:** Scene-stratified estimates of the Modified × Female interaction coefficient on positive facial valence.

Scene	${\hat{\beta }}_{\mathrm{Mod}\times F}$	Direction	Consistency with Pooled Estimate
A	−4.1	negative	✓
B	−5.2	negative	✓
C	−4.0	negative	✓
Pooled	−4.48	**negative**	—

**Table 7:** Means and Standard Deviations by Presentation Type.

Condition	Blink	Engag.	PosVal	NegVal	NeuVal	PosAdap	GSR PM
Original Color							
Mean	48.30	1.9617	1.4764	0.0096	91.9457	1.4264	3.2450
SD	45.97	4.4953	4.5042	0.0367	25.3684	4.4401	2.8390
*Black and White*							
Mean	50.23	1.6570	0.3593	0.0225	92.9447	0.3425	2.8703
SD	47.23	2.8367	1.1482	0.0724	25.2954	1.0984	3.0176
*Modified Color*							
Mean	41.87	3.6197	2.3732	0.0486	90.1747	2.2593	3.2243
SD	36.55	6.8277	5.7579	0.2003	25.4885	5.4017	3.6054

*Note.* Engagement values in this table correspond to raw engagement time expressed in seconds. In contrast, Table 3 reports normalized engagement indices derived from within-subject standardization. These representations are not directly comparable and serve different analytical purposes (descriptive vs. inferential).

## Data Availability

The datasets generated and analyzed during this study are publicly available in a public repository. The access link is provided as follows: https://drive.google.com/drive/u/0/folders/17_6QRcAtOdTMpWDC8BhO7PKgNWbOnZRV (accessed on 4 April 2026).

## Supplementary Materials

The following supporting information can be downloaded at: https://drive.google.com/drive/folders/17_6QRcAtOdTMpWDC8BhO7PKgNWbOnZRV (accessed on 4 April 2026).
